# Pherotypes are driving genetic differentiation within *Streptococcus pneumoniae*

**DOI:** 10.1186/1471-2180-9-191

**Published:** 2009-09-07

**Authors:** Margarida Carrolo, Francisco R Pinto, Jose Melo-Cristino, Mario Ramirez

**Affiliations:** 1 Instituto de Microbiologia, Instituto de Medicina Molecular, Faculdade de Medicina, Universidade de Lisboa, Av. Prof. Egas Moniz, 1649-028 Lisboa, Portugal; 2 Centro de Química e Bioquímica, Faculdade de Ciências, Universidade de Lisboa, Campo Grande, 1749-016 Lisboa, Portugal

## Abstract

**Background:**

The boundaries of bacterial species and the mechanisms underlying bacterial speciation are matters of intense debate. Theoretical studies have shown that recombination acts as a strong cohesive force preventing divergence in bacterial populations. *Streptococcus pneumoniae *populations have the telltale signs of high recombination with competence implicated as the major driving force behind gene exchange. Competence in *S. pneumoniae *is triggered by a quorum-sensing mechanism controlled by the competence-stimulating peptide pheromone.

**Results:**

We studied the distribution of the two major pherotypes in the pneumococcal population and their association with serotype, antimicrobial resistance and genetic lineage. Using multilocus sequence data we evaluated pherotype influence on the dynamics of horizontal gene transfer. We show that pherotype is a clonal property of pneumococci. Standard population genetic analysis and multilocus infinite allele model simulations support the hypothesis that two genetically differentiated populations are defined by the major pherotypes.

**Conclusion:**

Severe limitations to gene flow can therefore occur in bacterial species in the absence of geographical barriers and within highly recombinogenic populations. This departure from panmixia can have important consequences for our understanding of the response of pneumococci to human imposed selective pressures such as vaccination and antibiotic use.

## Background

Horizontal gene transfer and recombination, although recognized as important mechanisms in the evolution of certain phenotypes such as penicillin resistance in both *Neisseria meningitidis *and *Streptococcus pneumoniae*, were considered to be rare [1,2]. Full genome sequences and extensive surveys of bacterial populations using multilocus sequence typing (MLST) have challenged this view and established the essential role of horizontal gene transfer and recombination in bacterial evolution, revealing the high frequency of these events [3,4].

*Streptococcus pneumoniae *(pneumococcus) is an important human pathogen, taxonomically recognized as a group within the pneumoniae-mitis-pseudopneumoniae cluster of the *Streptococcus *genus [5]. The capacity of pneumococci to undergo genetic transformation was recognized early in the study of this bacterium [6] and it was later found that competence presented the intriguing property of being tightly controlled at the population level [7]. Competence was thus one of the first examples of a multicellular bacterial response coordinated by a diffusible signal. These processes were later termed quorum-sensing and found to be used by both Gram positive and Gram negative bacteria to synchronize the switch of genetic programs simultaneously at the population level in order to achieve goals that are unattainable by single cells [8]. Several molecules are used by bacteria to regulate their quorum-sensing mechanisms, with modified or unmodified oligopeptides being used by Gram positive and Gram-negative bacteria [8]. In *S. pneumoniae*, a secreted unmodified 17-aminoacid peptide pheromone, termed the competence-stimulating peptide (CSP), is responsible for quorum-sensing [9]. The product of the *comC *gene is secreted and processed by an ABC transporter (ComAB) resulting in the accumulation of CSP in the medium. A two-component regulatory system consisting of a histidine kinase receptor (ComD) and its cognate response regulator (ComE) are then responsible for sensing the CSP concentration and triggering the competence response.

In pneumococci several distinct mature CSPs have been identified, although the vast majority of strains produce one of two variants: CSP-1 or CSP-2 (also designated CSP-α and CSP-β, respectively) [5,10-12]. Each different CSP variant identifies a pherotype and strains genetically carrying one of the variants are mostly unable to respond to the presence of the other signaling peptide, possibly due to specificity of the ComD receptors for their cognate CSP alleles [10,11].

Competent bacteria will recognize and bind naked double stranded DNA fragments present in their environment, and translocate these fragments in a single stranded form across the membrane and into the cytoplasm. A number of genes facilitating recombination of the incoming DNA with the bacterial chromosome are also upregulated at competence, favoring the integration of the foreign DNA fragment that may permanently change the cell genotype and phenotype [9]. Competent cells are also endowed with the capacity to kill non-competent pneumococci in a mechanism named fratricide [13,14] and this may be a key property for transformation *in vivo *by providing a source of free DNA.

Pneumococcal fratricide is committed by cells that are competent and thus able to lyse non-competent siblings [13,15-17] with the concomitant release of DNA that will become available for transformation. The existence of two predominant pherotypes in *S. pneumoniae *and the documented occurrence of co-colonization [18,19], led to the proposal of two contrasting models of the pherotype impact on genetic exchange [15]. In the first model, the lack of inter-pherotype communication prevents genetic exchange between phenotypes favoring genetic differentiation [20,21]. The second model is based on the proposal that the absence of inter-pherotype cross-activation would result in a race for competence activation with the winning phenotype inducing the lysis of cells belonging to the other pherotype [22]. The latter would result in a more frequent exchange of genetic information between different pherotype lineages that is assumed to result in enhanced genetic diversity of pneumococci.

The human host is the only natural ecological niche of all pneumococcal strains where they are exposed to the same environmental insults and share very similar lifestyles. We propose that limitations to lateral gene transfer, through a kind of "assortative mating" promoted by the existence of two pherotypes, is creating genetically differentiated subpopulations within *S. pneumoniae*.

## Results and discussion

### Pherotype distribution among the pneumococcal population

Traditionally, pneumococcal strains have been characterized by their capsular polysaccharide (serotype) of which pneumococci produce 91 chemically and immunologically distinct variants [23]. Although it has been shown that the serotype defines important epidemiological and virulence properties of pneumococcal isolates [24], it is also recognized that each serotype comprises different clones that may present different properties [25].

The collection of 483 invasive pneumococcal isolates was characterized for the *comC *allele (pherotype) carried by each isolate. All isolates could be classified either as CSP-1 or as CSP-2 and, in agreement with previous findings, most presented the CSP-1 pherotype (70.6%) [see Additional file 1 - Table S1]. The data was analyzed to determine if the pherotypes were randomly distributed among the population or if there were associations with particular characteristics of the isolates, namely serotype, antibiotic resistance and the genetic lineages identified by pulsed-field gel electrophoresis (PFGE) profiling and MLST.

As a first approximation we used the Wallace coefficient (W) [26,27]. W provides an estimate of the probability of two strains sharing the same pherotype if they share another characteristic such as serotype or being classified in the same PFGE cluster. Table 1 shows the W values obtained, indicating that isolates sharing the same serotype have a high probability of belonging to the same pherotype (W = 0.730) and this probability is higher if the isolates belong to the same PFGE cluster (W = 0.771). Both values are significantly different from the expected values in case of a random association between pherotype and either of these two characteristics (W_i _= 0.584), demonstrating that pherotypes are not randomly dispersed within the pneumococcal population.

**Table 1:** Wallace's coefficients and respective confidence intervals testing the ability of several methods to predict the pherotype.

Parameter	W (95% CI)	W_i_^a^
Serotype	0.730 (0.689;0.772)	0.584
PFGE cluster	0.771 (0.726;0.816)	0.584
Sequence type	0.982 (0.964;1)	0.621
Clonal complex	0.986 (0.961;0.992)	0.621

^a^W_i _is the expected Wallace coefficient if the classification method is independent of the pherotype.

To determine if individual serotypes and PFGE clusters were significantly enriched in isolates presenting each pherotype, odds ratios (OR) were calculated. A total of five serotypes are significantly associated with either one of the pherotypes (Table 2 and see Additional file 1 - Table S1). The high Wallace values suggest that pherotype/serotype association is not only due to these five serotypes. Many serotypes are present in insufficient numbers to reach a significant odds ratio. By simultaneously looking at each pair of strains the Wallace statistic has an increased power to detect associations. Serotypes 1 and 14 are strongly associated with CSP-1 whereas serotypes 3, 6A and 9N show an association with CSP-2. The same approach was used to determine if pherotypes were associated with particular PFGE clusters within each serotype, aiming to subdivide serotypes into closely related genetic lineages. Five PFGE clusters showed association with a particular pherotype [see Additional file 2 - Table S2]. Of these, the largest PFGE clusters within serotypes 1, 3, 9N and 14 maintained the same association found between these serotypes and pherotype. Possibly due to the small number of isolates in each PFGE cluster, none of the clusters expressing serotype 6A was significantly associated with either pherotype, in contrast with the association found between this serotype and CSP-2. On the other hand, serotype 4 presents one PFGE cluster that was significantly associated with CSP-2, whereas no association was found at the serotype level possibly as a consequence of the largest cluster of serotype 4 being mainly CSP-1 [see Additional file 2 - Table S2]. Taken together the data suggest that pherotype is a clonal property that may vary independently of the serotype.

**Table 2:** Odds ratios measuring significant associations between pherotype and serotype.

Serotype	CSP-1	CSP-2	OR (95%CI)^a^	FDR^b^
1	48	2	11.434 (2.923;98.526)	< 10^-4^
3	23	23	0.375 (0.193;0.729)	0.017
6A	2	11	0.071 (0.007;0.330)	0.001
9N	2	8	0.099 (0.010;0.506)	0.013
14	61	4	7.497 (2.698;28.985)	< 10^-4^

^a^Odds ratio (OR) describes the strength of the association between a pherotype and a particular serotype. In each case, if the OR is significantly > 1, CSP-1 is associated with the serotype and if OR is significantly < 1 means that the serotype is enriched in CSP-2 beyond what would be expected.

^b^Values obtained after false-discovery rate correction for multiple testing

MLST is a sequence based approach that uses the sequence of internal fragments of housekeeping genes for the purpose of characterizing, typing, and classifying members of bacterial populations. The data derived from MLST can also be used to study the population genetics of bacteria such as *Streptococcus pneumoniae *[28]. Applying eBURST to MLST data originates subnetworks of isolates with increased probability of sharing a recent common ancestor. These subnetworks define clonal complexes as groups of isolates that share the alleles at no less than six loci with at least another member of that group [29]. MLST from 90 selected strains [30] revealed 57 different sequence types grouped into 39 distinct clonal complexes. The ability of sequence type and clonal complex to predict the pherotype is remarkably high, both with W > 0.97 (Table 1). PFGE and MLST are widely used tools to define bacterial clones, the fact that the groups defined by both these methods show such strong correspondence with pherotype further strengthen the indication that pherotype is a clonal property within the pneumococcal population.

A consistent hypothesis with pherotype clonality is that the role of CSP in triggering competence and its consequences on lateral gene transfer is itself responsible for the distribution of the pherotypes in the pneumococcal population. If this hypothesis is correct and the pherotype is indeed restricting gene transfer within the pneumococcal population, genes that are under recent strong selective pressure and that are known to be horizontally transferred should be associated with pherotype.

### Pherotype and antibiotic resistance

To test our hypothesis, we checked if there was an association between antibiotic resistance and pherotype. Resistance to several antibiotics in pneumococcus was shown to be mediated by the acquisition of foreign DNA that has subsequently spread within the pneumococcal population [31]. Emergence of resistance in pneumococci and its dissemination in the population is postulated to have occurred since their widespread use in clinical practice in the late 1940s. The results in Table 3 indicate that there was an association of most antibiotics (with the exception of erythromycin) with a particular pherotype. Isolates resistant to penicillin and other β-lactams were associated with CSP-1. It is known that resistance to β-lactams was acquired from closely related species of the mitis complex and that genes encoding resistance are transferred within the pneumococcal population by genetic recombination [31]. The fact that penicillin resistant isolates are more frequently CSP-1 suggests that, in addition to the expansion of resistant clones, current gene flow occurs primarily between isolates that share the same pherotype.

**Table 3:** Association between antibiotic resistance and pherotype.

Antibiotic	CSP-1		CSP-2		OR (95% CI)^a^	FDR^b^
	**Resistant**	**Susceptible**	**Resistant**	**Susceptible**		
Penicillin^c, d^	92	249	21	121	2.13 (1.24;3.78)	0.012
Erythromycin	32	309	16	126	0.82 (0.42;1.65)	0.611
Clindamycin	22	319	16	126	0.54 (0.26;1.15)	0.141
Tetracycline^d^	18	323	20	122	0.31 (0.16;0.70)	0.010
Chloramphenicol^d^	5	336	9	133	0.22 (0.05;0.75)	0.013
Co-trimoxazole^d^	89	252	17	125	2.59 (1.45;4.86)	0.005
Cefuroxime^d^	68	272	12	129	2.68 (1.38;5.64)	0.010

^a ^Odds ratio (OR) measures the strength of the association between a pherotype and resistance to a particular antibiotic. In each case, if OR is significantly > 1, CSP-1 is associated with resistance to that antibiotic and if OR is significantly < 1 this means that CSP-2 is associated with resistance to that particular antibiotic.

^b ^Correction for multiple testing performed by the false discovery rate method (FDR)

^c ^p < 0.05 after FDR correction.

^d ^Both penicillin intermediate and fully resistant isolates were considered resistant for this analysis.

### The relationship between pherotype and restriction/modification systems

Another important mechanism of lateral gene transfer is bacteriophage transduction [32]. This is an especially important mechanism for the transfer of large DNA fragments that may be restricted in transformation. This is for instance the case of the locus encoding the capsular polysaccharide biosynthesis machinery and of some of the genetic determinants of resistance to tetracycline, chloramphenicol or erythromycin, that are large composite transposons unable to transfer by conjugation, leaving phage transduction as the most likely mechanism of dissemination in the bacterial population, similarly to what was described in other streptococci [33].

Transduction should be independent of CSP activity, but the presence of restriction/modification (R/M) systems was shown to impair horizontal transfer through this mechanism [34]. Pneumococci are unusual in that they posses either one of two complementary R/M systems located in interchangeable genetic cassettes. Strains of *S. pneumoniae *contain either the *dpn*I cassete, containing an endonuclease that cleaves only the methylated DNA-sequence 5'GmeATC3' or the *dpn*II cassette, which includes an endonuclease that cleaves the same sequence when not methylated, together with the corresponding methylase. These mutually exclusive R/M systems were shown to protect against viral infection by viruses produced in cells of the opposite genotype, reducing infection frequency to < 10^-5 ^[35].

The R/M cassette has a size compatible with horizontal transfer by transformation, so we wondered if the distribution of the R/M cassettes could be correlated to the pherotype and thereby contribute to promote asymmetries of horizontal gene transfer within the pneumococcal population.

To pursue this hypothesis, the R/M cassette carried by pneumococcal isolates previously characterized by MLST was determined. The proportion of CSP-2 isolates with the *dpn*II cassette (3/23) is lower than the proportion of CSP-1 isolates with that same cassette (25/67) and the association between pherotype and the R/M system is significant (p = 0.037, Fisher exact test), suggesting that phage transduction may be indirectly arbitrated by the pherotype *via *the R/M systems, such that the spread of large genetic elements that rely on this mechanism of horizontal gene transfer could also be limited by pherotype.

### Pherotype is a marker of population segregation

MLST data has been used to characterize the clonality of bacterial populations and to explore the impact of recombination and mutation in bacterial evolution [4]. For *S. pneumoniae *the recombination rate has been estimated to be 3-10 times the mutation rate per locus [28,36]. To test if the pherotype could be limiting the genetic exchanges within pneumococci, we took the simple approach of testing among all pairs of sequence types that diverge at the allele of a single locus (single-locus variants - SLV) and that should represent the initial stages of diversification dominated by recombination, if the allele that differed was more frequent among sequence types sharing the same pherotype or among isolates of a different pherotype. Considering the observed SLV pairs in our study, the probability that the changing allele came from a different pherotype is 0.11. In a panmictic population, the expected probability would be 0.38 (p < 10^-4^, permutation test), again suggesting that recombination between pherotypes is reduced.

To test if the populations defined by each pherotype showed genetic differentiation we analyzed the concatenated sequences of six of the genes used in MLST, excluding *ddl *since it was previously shown that this gene showed a hitchhiking effect with *pbp2b *involved in penicillin resistance[37] and could thus bias the results. Out of 143 mutations in 142 polymorphic sites, 66 were shared between the two pherotype defined populations, 63 were polymorphic in the CSP-1 population but monomorphic in the CSP-2 population and 14 were polymorphic in CSP-2 but monomorphic in CSP-1. To estimate the level of gene flow and whether pherotype defined diverging populations, the classic F_ST _parameter [38], the K*_ST _statistic [39] and the more powerful nearest-neighbor statistic S_nn _[40] were used. The F_ST_, K*_ST _and S_nn _statistics are measures of population differentiation based on the number of differences between haplotypes. The statistical significance of both the K*_ST _and S_nn _statistics were evaluated by permutation. The data in Table 4 shows that statistically significant K*_ST _values (p < 0.01) were obtained not only for the analysis of the concatenated sequences but also for most of the individual genes. The more sensitive S_nn _statistic presented significant values (p < 0.01) for the analysis of the concatenated sequence as well as for all individual genes.

**Table 4:** Nucleotide variation and population differentiation parameters.

Alleles	π	F_ST_	K*_ST_	p (K*_ST_)^a^	S_nn_	p (S_nn_)^a^
*aroE*	0.005	0.021	0.018	0.022	0.721	< 10^-4^
*gdh*	0.009	0.025	0.008	0.115	0.706	0.004
*gki*	0.019	0.134	0.045	< 10^-4^	0.810	< 10^-4^
*recP*	0.005	0.072	0.039	0.001	0.717	< 10^-4^
*spi*	0.009	0.190	0.062	< 10^-4^	0.677	0.004
*xpt*	0.007	0.133	0.042	< 10^-4^	0.790	< 10^-4^
*ddl*	0.012	0.018	0.012	0.033	0.738	< 10^-4^
Combined^b^	0.009	0.115	0.025	< 10^-4^	0.833	< 10^-4^

^a^Probabilities evaluated by 1,000 permutations.

^b^The results correspond to the analysis of the concatenated sequences of the *aroE*, *gdh*, *gki*, *recP*, *spi *and *xpt *alleles.

**Figure 1:**
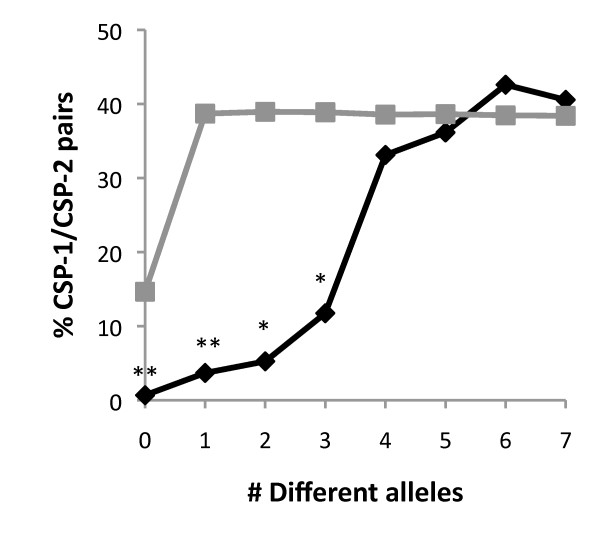
**Probability of pairs of isolates with different alleles to belong to different pherotypes**. The black line indicates the fraction of observed CSP-1/CSP-2 pairs differing at the indicated number of alleles and the grey line the expected number if there was a random association between pherotype and sequence type. As the allelic differences increase, the probability of diverging in pherotype also increases reaching levels undistinguishable from those expected by chance when strains differ in more than three alleles. One asterisk, p < 0.01 and two asterisks, p < 0.001.

A different approach to test if the pherotype is a marker of genetic isolation consists of calculating the probability that pairs of isolates with increasing levels of genetic divergence have of belonging to different pherotypes. Figure 1 shows that the closest pairs of isolates have a significantly lower probability of having different pherotypes. When genetic divergence increases, the probability of differing in pherotype also increases, reaching the levels expected by chance when isolates differ in more than three alleles. Again, these results show that isolates that are phylogenetically closely linked have an increased likelihood of sharing the same pherotype.

### Infinite allele model

**Figure 2:**
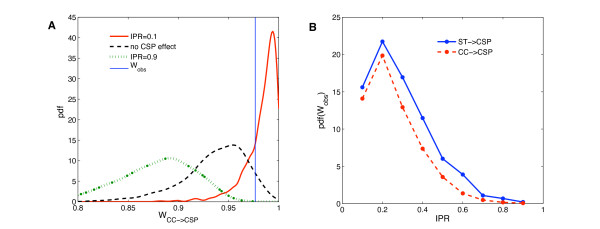
**Probability density function of Wallace values for simulated populations**. Multilocus sequence types of a pneumococcal population were generated with an adapted infinite allele model [36]. It includes an additional locus for CSP type and a new parameter IPR that, given a recombination event, defines the probability that the two recombining strains have different pherotypes. The prevalence of each pherotype in the population was fixed during the simulation at 70% for CSP-1 and 30% for CSP-2. (A) From 1,000 simulations, the probability density functions of Wallace values for Clonal Complex predicting pherotype were computed for three scenarios: (1) pherotype is a barrier to recombination (IPR = 0.1, red line), (2) pherotype has no impact in gene exchange (equivalent to IPR = 0.42, black dashed line) and (3) inter-pherotype recombination is favored (IPR = 0.9, green dotted line). The vertical blue line indicates the observed Wallace value in the studied sample. (B) To identify the value of the IPR parameter that is in best agreement with the data, the probability density at the observed Wallace values was computed for simulated populations with varying inter-pherotype recombination probabilities (IPR from 0.1 to 0.9), both for Wallace indexes of sequence type (blue line) and of clonal complex (red line) predicting pherotype.

The structured nature of the pneumococcal population and the geographically limited origin of our sample could explain, at least partially, the segregation of pherotypes seen in Figure 1 and the high Wallace indices of Table 1. To address this issue a MLST infinite allele model was used to test the effect of restricting or promoting recombination between the two pherotype defined subpopulations. This modeling approach was previously shown to reproduce the clonal structure of the pneumococcal population [36,41] and provides a possibly more realistic null hypothesis for the distribution of phenotypes in the population. The model was expanded to include a new locus with two possible alleles: CSP-1 and CSP-2. This extra locus recombines with the same rate as the MLST loci and the frequency of each allele is kept constant and equal to 70 and 30% of CSP-1 and CSP-2 respectively, corresponding to the observed values in natural populations. Additionally, a new parameter IPR was introduced, that controls the probability of inter-pherotype recombination. If pherotype differences would not prevent or promote recombination, the observed frequencies of each pherotype in the population would lead to a probability of inter-pherotype recombination of 0.42. Figure 2A shows that even in the absence of a pherotype effect on recombination, high Wallace values of clonal complex predicting pherotype are expected. This result is intuitive since the recent common ancestry of strains belonging to the same clonal complex would also cause them to share the same pherotype. Still, there is a marked shift to higher Wallace values when the probability of inter-pherotype recombination decreases (IPR = 0.1 in Figure 2A). On the other hand, if genetic exchange between pherotypes is favored, in spite of their different prevalence in the population (IPR = 0.9 in Figure 2A), a shift towards lower W_CC→ST _values is observed. When systematically varying IPR and computing the probability density for the observed Wallace coefficients (Figure 2B), one concludes that a value of 0.2 is 2-3 times more likely to explain the observed values than an IPR of 0.42, expected in case of no CSP effect in recombination. Since the more probable IPR is lower than expected if the two pherotype populations were recombining freely, these results strengthen the proposal that recombination is promoted within individuals sharing the same pherotype, promoting the divergence of two subpopulations of *S. pneumoniae*.

## Conclusion

In agreement with previous suggestions [14,20,21], we propose that the specific ComC/ComD match facilitates a form of assortative genetic exchange, which could maintain genetically diverse subpopulations within this species. Although recent studies addressing the phenomenon of fratricide in pneumococci favor the hypothesis of preferential inter-pherotype genetic exchange [42], the data presented here argues that in natural populations intra-pherotype exchanges prevail, creating a barrier to gene exchange. In vitro studies that led to the fratricide hypothesis show that if two pneumococcal strains with different pherotypes are grown together, the one that becomes competent earlier will have a greater probability of being transformed with DNA from the other strain [42]. In order to observe the impact of this admixture promoting event in pneumococcal natural populations, frequent and adequate co-colonization events involving different pherotypes must occur. On the other hand, fratricide has also been observed in experiments with a single strain [13]. Dynamic bi-stable regulatory systems, as described for *Bacillus subtilis *[43], may underlie the mechanism leading to the simultaneous presence of competent and non-competent cells of the same strain or the same pherotype. If natural co-colonization by strains of different pherotypes is rare or inadequate to promote gene exchange, it is possible to reconcile the inter-pherotype fratricide observations with the pherotype defined genetic differentiation identified here. The observed genetic barrier would then be justified if co-colonization events involving different strains of the same pherotype are more frequent or more adequate for recombination, leaving intra-pherotype fratricide and genetic exchange as the most common event in natural populations.

All the isolates analyzed were recovered in Portugal from invasive infections and it is therefore unlikely that geographic or ecological fragmentation could explain the pattern observed. The model simulations also exclude the possibility that our observation results simply from the structure of the pneumococcal population, with multiple isolates sharing the same genotype or with a recent common ancestry. It would also be plausible to assume that the CSP-2 population was recently established by introduction of a novel pherotype into pneumococci. This would result in the genome wide differentiation observed, but if it had occurred recently it would also cause lower haplotype diversity in the CSP-2 population, that the data does not support. Furthermore, the CSP-2 pherotype was found in multiple serotypes and clones, including strains differing in the alleles of up to five of the seven genes used in the pneumococcal MLST scheme. These observations support an ancient origin of the CSP-2 pherotype that would have allowed sufficient time for the coalescence of the two pherotype defined populations due to the high recombination of pneumococci.

Although only invasive strains were used in the present study, a comparison of previous studies [30,44] indicates that clones found causing invasive infections are also found among the most prevalent in carriage, meaning that the results described here are also expected to be valid for the overall pneumococcal population in Portugal.

The concept of allopatric speciation follows the intuitive rationale that genetic divergence subsequent to geographic isolation could lead to the emergence of different species [45]. In bacteria, this has been connected with the concept of ecotypes [46], arising as a consequence of a single clone expanding into a new niche. These events have been implicated in the emergence of human pathogens from environmental or commensal species, such as the rise of *Yersinia pestis *or *Mycobacterium tuberculosis *from within the *Yersinia *and mycobacteria respectively [47]. But genetic differentiation in microorganisms was also shown to occur mainly as a result of geographic barriers, such as that of the wild yeast *Saccharomyces paradoxus *[48].

In the absence of ecological isolation, a process of sympatric speciation, shown to occur in sexual eukaryotes [45], is deemed unlikely in bacteria due to the occurrence of recombination. In fact, theoretical studies have shown that if recombination is more frequent than mutation, the "cohesive force of recombination" is an effective barrier to divergence and to bacterial speciation [49,50]. This received further support from the recent observation of an accelerated convergence of species within the *Campylobacter *genus proposed to be caused by the breakdown of ecological or geographical barriers and the effect of recombination [51].

Pneumococci are generally considered a sexual population due to the dominant role of recombination in the evolution of this species [49]. It was therefore surprising to find that two genetically distinct subpopulations could be identified. Extensive sequence divergence, previously shown to be a major barrier to gene exchange [52], could not be implicated as attested by the low π values and the fact that 66 out of the 143 mutations were shared between the two pherotype populations. Interestingly, the existence of three differentiated subpopulations within pneumococci, with different rates of admixture, was recently inferred using a Bayesian method of population analysis [53], but no explanation for this differentiation was presented.

We propose that "assortative mating" mediated by different pherotypes and ongoing genetic drift may be driving an incipient speciation process within *S. pneumoniae*. Our data support theoretical predictions that the existence of barriers to recombination allow the accumulation of significant genetic drift, even within highly recombinogenic bacterial species. An understanding of these mechanisms and their consequences offer further insights into the evolution of bacterial pathogens and may allow more informed predictions on the consequences of human interventions such as antibiotic use and vaccination on bacterial populations.

### Addendum in proof

We recently became aware of a study (Omar Cornejo, personal communication) that has addressed the same issue discussed here. In contrast to our findings, the authors failed to detect any differentiation between the two pherotype defined populations. The reasons behind this discrepancy of results is not clear and further studies are needed to reconcile these apparently contradictory findings.

## Methods

### Bacterial strains, growth conditions, PFGE and MLST

A collection of 483 invasive pneumococcal isolates recovered during the period of 1999 to 2002 in Portugal were obtained from the Faculdade de Medicina de Lisboa collection. The serotype, PFGE type, MLST characterization and antibiotic susceptibility of these strains were collected from previous studies[25,30,54]. Briefly, all *S. pneumoniae *strains were grown in a casein-based semi-synthetic medium (C+Y) at 37°C without aeration or in tryptic soy agar (TSA) (Oxoid, Hampshire, England) supplemented with 5% (v/v) sterile sheep blood incubated at 37°C in 5% CO_2_. Antimicrobial susceptibility, serotyping and PFGE analysis was performed for all isolates. MLST analysis was performed for at least one isolate in each major PFGE cluster (n = 90) and revealed 57 different sequence types (ST) corresponding to 39 different lineages by eBURST analysis.

### Detection of the pherotype and endonuclease restriction phenotype by PCR

CSP-1 and CSP-2 gene fragments were amplified using multiplex PCR with primers CSP_up (5'-TGA AAA ACA CAG TTA AAT TGG AAC-3'), CSP1_dn (5'-TCA AGA AAG GAT AAA GGT AGT CCT C-3') and CSP2 _dn (5'-TAA AAA TCT TTC AAT CCC TAT TT-3'), which allowed the amplification of fragments of 620 bp for the CSP-1 allele and 340 bp for the CSP-2 allele. *dpn*I and *dpn*II genotype was also detected by multiplex PCR with primers DpnI_up (5'-GAA GTA GGA GAT AAA TTG CCA GAG), DpnII_up (5'-TAC GAA TGA TGG GAA TAC TGT G-3') and Dpn_dn (5'-TGT CCT CAA TGC CGT ATT AAA TC-3'), with the expected products of 342 bp and 421 bp for *dpn*I and *dpn*II, respectively. Template DNA was prepared by diluting 9 μl of an overnight culture in 441 μl of water and boiling this mixture for 2 minutes. The PCR reactions were performed in 50 μl of final volume containing 20 μl of template solution, 1× reaction buffer (Biotools, Madrid, Spain), 10 mM dNTPs (Fermentas, Vilnius, Lithuania), 20 pmol of each of the primers and 1.25 U GoTaq Polymerase (Invitrogen, Carlsbad, California). The same PCR program was used consisting of 30 cycles of denaturation at 95°C for 1 min, annealing at 55°C for 30 sec, and primer extension at 72°C for 1 min. Followed by 10 min incubation at 72°C to complete extension.

### Data analysis

Statistical association between serotypes, PFGE clusters, antimicrobial resistance or endonuclease restriction phenotype and pherotype where characterized by odds ratios (OR) with 95% confidence intervals (CI) computed through the Fisher method implemented in the epitools package for the R language. OR significance was evaluated with the Fisher exact test. The resulting p-values were corrected for multiple testing by controlling the False Discovery Rate (FDR) under or equal to 0.05 through the linear procedure of Benjamini and Hochberg [55].

Wallace coefficients (W) and respective 95% confidence intervals were computed as previously described [26,27].

The relationship between cross-pherotype pair frequency and the number of divergent alleles between STs was validated for statistical significance by permutation tests. The latter consisted in repeating the computation of frequencies of cross-pherotype strain pairs for 1,000 times, randomly shuffling the pherotype assignment of the strains before each repetition. The p-values were obtained from the fraction of the 1,000 random runs where the cross-pherotype pair frequency was lower than the respective values with the correct pherotype assignment. A permutation test was also performed to evaluate the significance of the probability that a divergent allele in an SLV pair was donated from a strain with a different pherotype. In this case, in each of the 1,000 runs, the divergent allele was randomly sampled from the corresponding locus in the collection of STs. The determination of π, F_ST_, K*_ST _and S_nn _for the analysis of sequence data was done using the DNASP v4.50.3 program. The values of K*_ST _and S_nn _were used to assess population differentiation in combination with permutation tests (1,000 permutations).

### Neutral Multilocus Infinite Allele Model

The model presented by Fraser et al. [36] was expanded to include an additional CSP locus and a new IPR parameter. The CSP locus has only two possible alleles, CSP-1 and CSP-2 that can interchange by recombination but are not affected by mutations. The parameter IPR defines the inter-pherotype recombination probability. The model was simulated with the parameter values determined in [36] for the pneumococcal population. Namely, the population size was 1,000, the population mutation and recombination rates were 5.3 and 17.3, respectively. All the analyses were repeated with a population recombination rate reduced in 50% and the results were qualitatively similar. All simulations were run for 1,000 generations, after which the sequence type diversity was stable, as measured by the Simpson's index of diversity [56]. At each generation, 70% of the selected individuals were CSP-1 and 30% were CSP-2. For each value of parameter IPR, 1,000 independent simulations were carried out. Wallace coefficients for ST and CC predicting CSP type were calculated for each of the final 1,000 populations. Probability density functions for the Wallace distributions were determined by kernel density estimation with a Gaussian kernel function. All simulations and computations were done in Matlab version 7.7.

## Authors' contributions

MC, FRP, JMC and MR designed research; MC performed research; FRP and MR analyzed data; MC, FRP, JMC and MR wrote the paper. All authors read and approved the final manuscript.

## Supplementary Material

Additional file 1 **Table S1 - Pherotype distribution arranged by serotype in the pneumococcal collection**. Odds ratios (OR) represent the strength of the association between a pherotype and a particular serotype. In each case, if the OR is significantly > 1, CSP-1 is associated with the serotype and if OR is significantly < 1 means that the serotype is enriched in CSP-2. Click here for file

Additional file 2 **Table S2 - Pherotype distribution arranged by PFGE cluster in the pneumococcal collection**. Odds ratios (OR) represent the strength of the association between a pherotype and a particular PFGE cluster. In each case, if the OR is significantly > 1, CSP-1 is associated with the PFGE cluster and if OR is significantly < 1 means that the PFGE cluster is enriched in CSP-2. Click here for file
