# Supplementary material for: Pherotypes are driving genetic differentiation within Streptococcus pneumoniae
Source: BMC Microbiol. 2009 Sep 7;9:191. doi: 10.1186/1471-2180-9-191 (PMC2751782; doi:10.1186/1471-2180-9-191)
Supplement: Additional file 1 — Table S1 - Pherotype distribution arranged by serotype in the pneumococcal collection. Odds ratios (OR) represent the strength of the association between a pherotype and a particular serotype. In each case, if the OR is significantly > 1, CSP-1 is associated with the serotype and if OR is significantly < 1 means that the serotype is enriched in CSP-2. [file 1471-2180-9-191-S1.pdf]

Table S1. Pherotype distribution among serotypes.

Serotype	CSP-1	CSP-2	OR (95%CI)	FDR[a]
1[b]	48	2	11.43 (2.93;98.52)	$<10^{-4}$
3 [b]	23	23	0.38 (0.19;0.73)	0.017
4	16	13	0.49 (0.21;1.14)	NS[c]
5	9	4	0.94 (0.26;4.23)	NS[c]
6A [b]	2	11	0.08 (0.007;0.33)	$<10^{-4}$
6B	9	5	0.74 (0.22;2.88)	NS[c]
7C	0	1	0 (0;16.24)	NS[c]
7F	14	4	1.48 (0.45;6.27)	NS[c]
8	20	8	1.04 (0.43;2.81)	NS[c]
9L	0	1	0 (0; 16.24)	NS[c]
9N [b]	2	8	0.10 (0.01;0.51)	0.013
9V	20	2	4.35 (1.03;38.92)	NS[c]
10A	4	5	0.33 (0.064;1.54)	NS[c]
10F	2	1	0.83 (0.04;49.42)	NS[c]
11A	7	0	Inf[d] (0.60;Inf[d])	NS[c]
11B	0	1	0 (0;16.24)	NS[c]
12B	13	3	1.83 (0.49;10.19)	NS[c]
13	2	1	0.83 (0.04;49.42)	NS[c]
14 [b]	61	4	7.50 (2.70;28.99)	$<10^{-4}$
15B	2	0	Inf[d] (0.08;Inf[d])	NS[c]
16F	2	2	0.41 (0.00;5.76)	NS[c]
17F	0	2	0 (0;2.21)	NS[c]
18A	3	2	0.62 (0.07;7.52)	NS[c]
18C	8	4	0.83 (0.22;3.83)	NS[c]
19A	12	5	1.00 (0.32;3.69)	NS[c]
19F	5	6	0.34 (0.08;1.35)	NS[c]
20	3	2	0.62 (0.07;7.52)	NS[c]
22F	12	0	Inf[d] (1.18;Inf[d])	NS[c]
23A	1	0	Inf[d] (0.01; Inf[d])	NS[c]
23B	2	0	Inf[d] (0.08; Inf[d])	NS[c]
23F	18	6	1.26 (0.47;3.97)	NS[c]
24F	0	2	0 (0;2.21)	NS[c]
25A	0	1	0 (0;16.24)	NS[c]
25F	0	1	0 (0;16.24)	NS[c]
31	0	3	0 (0; 1)	NS[c]
33A	1	0	Inf[d] (0.01;Inf[d])	NS[c]
33F	7	0	Inf[d] (0.60;Inf[d])	NS[c]
35B	1	0	Inf[d] (0.01;Inf[d])	NS[c]
35F	3	0	Inf[d] (0.17;Inf[d])	NS[c]
36	0	1	0 (0;16.24)	NS[c]
38	1	1	0.42 (0.005;32.78)	NS[c]
NT[e]	8	7	0.42 (0.14;1.54)	NS[c]

[a] Correction for multiple testing performed by the false discovery rate method (FDR)
[b] $p<0.05$ after FDR correction
[c] NS, not significant
[d] Inf, infinite value.
[e] NT, non-typable strains
