# Supplementary material for: Pherotypes are driving genetic differentiation within Streptococcus pneumoniae
Source: BMC Microbiol. 2009 Sep 7;9:191. doi: 10.1186/1471-2180-9-191 (PMC2751782; doi:10.1186/1471-2180-9-191)
Supplement: Additional file 2 — Table S2 - Pherotype distribution arranged by PFGE cluster in the pneumococcal collection. Odds ratios (OR) represent the strength of the association between a pherotype and a particular PFGE cluster. In each case, if the OR is significantly > 1, CSP-1 is associated with the PFGE cluster and if OR is significantly < 1 means that the PFGE cluster is enriched in CSP-2. [file 1471-2180-9-191-S2.pdf]

Table S2. Pherotype distribution among PFGE clusters.

Serotype	no. of isolates	PFGE cluster	CSP-1	CSP-2	OR (95%CI)	FDR[a]
1	50	A[b]	32	1	14.56 (2.38;597.17)	0.012
		B	16	1	6.93 (1.06;292.78)	0.317
3	46	A[b]	20	22	0.34 (0.17;0.68)	0.029
		B	3	0	Inf[c] (0.17; Inf[c])	0.948
		C	0	1	0 (0;16.24)	0.593
4	29	A	2	0	Inf[c] (0.08; Inf[c])	1
		B	0	3	0 (0;1)	0.293
		C[b]	2	9	0.09 (0.01;0.43)	0.016
		D	12	1	5.13 (0.75;221.18)	0.589
6A	13	A	0	1	0 (0;16.24)	0.593
		B	0	1	0 (0;16.24)	0.593
		C	0	3	0 (0;1)	0.293
		D	0	1	0 (0;16.24)	0.593
		E	1	1	0.42 (0.005;32.78)	0.877
		F	0	2	0 (0;2.21)	0.457
		G	0	1	0(0;16.24)	0.593
		H	1		Inf[c] (0.01; Inf[c])	1
		I	0	1	0 (0;16.24)	0.593
9N	10	A[b]	2	8	0.10 (0.01;0.51)	0.029
14	65	A	2	0	Inf[c] (0.08; Inf[c])	1
		B	10	0	Inf[c] (0.94; Inf[c])	0.373
		C	2	0	Inf[c] (0.08; Inf[c])	1
		D[b]	46	3	7.21 (2.25;36.84)	0.006
		E	1	1	0.42 (0.005;32.78)	0.877

[a] Correction for multiple testing performed by the false discovery rate method (FDR)
[b] p<0.05 after FDR correction.
[c] Inf, infinite value.
